# Reactivation of Plasma Butyrylcholinesterase by Pralidoxime Chloride in Patients Poisoned by WHO Class II Toxicity Organophosphorus Insecticides

**DOI:** 10.1093/toxsci/kft217

**Published:** 2013-09-19

**Authors:** Lisa A. Konickx, Franz Worek, Shaluka Jayamanne, Horst Thiermann, Nicholas A. Buckley, Michael Eddleston

**Affiliations:** *Department of Pharmacology, Toxicology, and Therapeutics, University/BHF Centre for Cardiovascular Science, University of Edinburgh, Edinburgh, EH16 4TJ, UK;; †School of Pharmacy, University of Utrecht, 3508 TC Utrecht, The Netherlands;; ‡Bundeswehr Institute of Pharmacology and Toxicology, 80937 Munich, Germany;; §Faculty of Medicine, University of Kelaniya, Colombo, Sri Lanka;; ¶South Asian Clinical Toxicology Research Collaboration, University of Peradeniya, Sri Lanka; and; ||Medical Professorial Unit, Prince of Wales Hospital Clinical School, University of New South Wales, Sydney, NSW 2052, Australia

**Keywords:** organophosphorus insecticides, butyrylcholinest erase, pralidoxime, marker, human poisoning.

## Abstract

Some clinicians assess the efficacy of pralidoxime in organophosphorus (OP) poisoned patients by measuring reactivation of butyrylcholinesterase (BuChE). However, the degree of BuChE inhibition varies by OP insecticide, and it is unclear how well oximes reactivate BuChE *in vivo*. We aimed to assess the usefulness of BuChE activity to monitor pralidoxime treatment by studying its reactivation after pralidoxime administration to patients with laboratory-proven World Health Organization (WHO) class II OP insecticide poisoning. Patient data were derived from 2 studies, a cohort study (using a bolus treatment of 1g pralidoxime chloride) and a randomized controlled trial (RCT) (comparing 2g pralidoxime over 20min, followed by an infusion of 0.5g/h, with placebo). Two grams of pralidoxime variably reactivated BuChE in patients poisoned by 2 diethyl OP insecticides, chlorpyrifos and quinalphos; however, unlike acetylcholinesterase reactivation, this reactivation was not sustained. It did not reactivate BuChE inhibited by the dimethyl OPs dimethoate or fenthion. The 1-g dose produced no reactivation. Pralidoxime produced variable reactivation of BuChE in WHO class II OP-poisoned patients according to the pralidoxime dose administered, OP ingested, and individual patient. The use of BuChE assays for monitoring the effect of pralidoxime treatment is unlikely to be clinically useful.

## INTRODUCTION

Organophosphorus (OP) insecticide self-poisoning is a major global health problem (Bertolote *et al.*, 2006; Jeyaratnam, 1990), with hundreds of thousands of deaths each year in rural regions of the developing world (Eddleston, 2000; Gunnell *et al.*, 2007). Although the more toxic World Health Organization (WHO) class I OPs (those with rat oral LD_50_s of less than 50mg/kg; World Health Organization, 2010) are being removed from agricultural practice, WHO class II OPs (with rat oral LD_50_ of 50mg/kg or more) are still widely used.

OP insecticides inhibit the enzymes acetylcholinesterase (AChE, EC 3.1.1.7) and butyrylcholinesterase (BuChE, EC 3.1.1.8), although some require metabolism to an oxon form via cytochrome P450 enzymes before becoming active (Lotti, 2001). Clinical features arise from inhibition of AChE causing overstimulation at cholinergic synapses in the autonomic nervous system, neuromuscular junction, and central nervous system (Ballantyne and Marrs, 1992). BuChE inhibition, in contrast, appears not to result in clinical features (Ballantyne and Marrs, 1992; Lotti, 2001). Management (Eddleston *et al.*, 2008a; Johnson *et al.*, 2000) involves resuscitation and the administration of the muscarinic antagonist atropine (Heath and Meredith, 1992) and an oxime AChE reactivator such as pralidoxime (Eyer, 2003). The beneficial effects of atropine are clear (Freeman and Epstein, 1955; Heath and Meredith, 1992; Johnson *et al.*, 2000). By contrast, the effect of pralidoxime remains unclear (Buckley *et al.*, 2011).

Clinicians have used reactivation of cholinesterase activity in blood as a way of measuring the effect of pralidoxime treatment in poisoned patients. Red-cell AChE assays should be more reliable because clinical effects result from synaptic AChE inhibition, and red-cell AChE has a close kinetic similarity with synaptic AChE. However, assays for BuChE activity are widely available and routinely performed and can be done on routinely sampled plasma samples, whereas AChE assays require whole blood samples that are rapidly cooled (Eyer, 2003). Therefore, BuChE assays have been used by some physicians to grade severity and to assess reactivation and pralidoxime efficacy (Abdullat *et al.*, 2006; Khan *et al.*, 2001; Kwong, 2002; Lee and Tai, 2001; Namba *et al.*, 1971; Pham, 2007).

Using BuChE for this purpose is complicated by the variability in BuChE inhibition and perhaps BuChE reactivation by pralidoxime, according to the OP (Eddleston et al., 2005, 2008b). *In vitro* studies have reported mixed results as to whether pralidoxime can reactivate BuChE inhibited by WHO class I OPs (Aurbek *et al.*, 2009; Jafari and Pourheidari, 2006; Musilova *et al.*, 2009). In addition, BuChE becomes aged after inhibition by both diethyl and dimethyl OPs so that it becomes unresponsive to reactivation by pralidoxime (Aurbek *et al.*, 2009).

It is, therefore, currently unclear whether measurement of BuChE activity might be appropriate for monitoring pralidoxime treatment in class II OP-poisoned patients. The aim of this study was to examine the reactivation of BuChE *in vivo* after pralidoxime treatment in Sri Lankan patients with laboratory-proven WHO class II OP insecticide poisoning. The data used were derived from 2 published studies: an observational cohort study (Eddleston *et al.*, 2005) and a randomized controlled trial (RCT) (Eddleston *et al.*, 2009a) using 2 different regimens of pralidoxime chloride. Analysis of these 2 studies has not previously assessed the effect of pralidoxime on reactivation of BuChE.

## MATERIALS AND METHODS

Institutional Review Board approval was received from the Faculty of Medicine Ethics Committee, Colombo, and Oxfordshire Clinical Research Ethics Committee. The RCT was established in response to systematic reviews (Buckley *et al.*, 2005; Eddleston *et al.*, 2002) that showed a lack of evidence for pralidoxime effectiveness and has been published in full (Eddleston *et al.*, 2009a). The results by RCT indicated that pralidoxime was not effective; as a result, pralidoxime was rejected by the WHO’s Essential Drugs List (World Health Organization, 2009), and an updated Cochrane systematic review (http://www.cochrane.org/cochrane-reviews) has reported a lack of evidence for effectiveness (Buckley *et al.*, 2011).

Written informed consent was taken from each patient, or their relatives (for patients unconscious or under the age of 16), in their own language.

### 

#### 

##### Cohort study.

Patients were identified on admission to 3 Sri Lankan hospitals between March 31, 2002, and May 25, 2004, to observe the difference in clinical features and severity of poisoning for the most common OP insecticides (Eddleston *et al.*, 2005). The patients received atropine according to a standard protocol (Eddleston *et al.*, 2004) and pralidoxime chloride as a 1-g bolus followed by further 1-g bolus doses every 6h for 1–3 days.

##### Randomized controlled trial.

The RCT was conducted in Anuradhapura and Polonnaruwa district hospitals in Sri Lanka from May 26, 2004, until October 18, 2006, to compare the effectiveness of pralidoxime treatment with placebo, in addition to standard therapy, in OP insecticide poisoning (Eddleston *et al.*, 2009a). Patients were randomized to 2 study arms to receive saline placebo or pralidoxime chloride. Pralidoxime was given as a 2-g loading dose over 20min, followed by an infusion of 0.5g/h until a maximum of 7 days, the patients no longer required atropine, or death.

For both studies, blood samples were taken from patients before and after pralidoxime administration to measure plasma BuChE activity and pralidoxime and OP insecticide concentrations. Sampling and assays were carried out as described (Eyer, 2003; Worek *et al.*, 1999). The mean control AChE and BuChE values in the assay are 586 (SD 5) mU/mmol Hb and 5932 (SD 33) mU/ml, respectively (Worek *et al.*, 1999). The lower boundary of normal BuChE was set at 3000 mU/ml.

##### Patient eligibility.

For this analysis, we included patients from both studies who showed biochemical evidence of cholinergic poisoning (BuChE activity less than 3000 mU/ml) for whom we had proof of the OP ingested and both prepralidoxime and postpralidoxime blood sample analyses. Exclusion criteria were ingestion of more than 1 OP insecticide, incomplete data files, and a different pralidoxime treatment regimen.

##### Statistical analysis.

The data analysis was performed in GraphPad Prism (version 5). For both cohort and the RCT, cholinergic activities were summarized with counts (percentages) for categorical variables, and the median (interquartile range [IQR]) for continuous variables, as none were expected to be normally distributed. BuChE activity at baseline for each agent was compared using the Kruskal-Wallis test. BuChE reactivation from baseline to 1h postpralidoxime was assessed overall and for each agent using a 2-sided paired *t* test and reported as the difference (95% confidence interval [CI] of the difference). (Due to the relatively small size of the sample, tests of normality were not performed. Sensitivity analyses using the nonparametric equivalent of the paired *t* test showed no substantial difference.)

## RESULTS

**FIG. 1.**
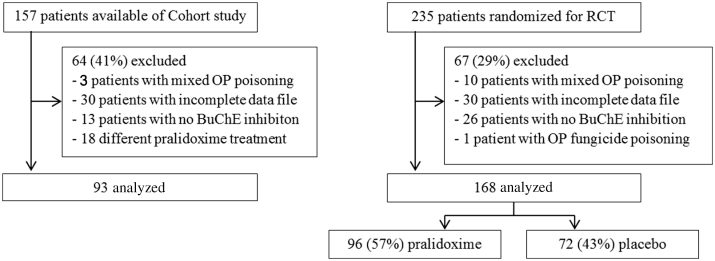
Flow diagram of progress through cohort and RCT data. Abbreviations: BuChE, butyrylcholinesterase; RCT, randomized controlled trial.

Patients were selected from the 2 study databases. Of the 802 patients in the published cohort study, only 157 had BuChE measurements performed (Fig. 1). Ninety-three patients met the inclusion criteria (Tables 1 and 2). The RCT randomized 235 patients, of whom 168 met the inclusion criteria for this study. Ninety-six patients were treated with pralidoxime and 72 with placebo (Tables 1 and 2).

**TABLE 1:** Distribution of OP Insecticide Ingested by Each Patient

Number	Cohort	RCT Pralidoxime	RCT Placebo
	*n* = 93	*n* = 96	*n* = 72
Organophosphorus insecticide ingested, *n* (%)			
Chlorpyrifos	71 (76.3)	51 (53.1)	35 (48.6)
Quinalphos	5 (5.4)	5 (5.2)	5 (6.9)
Dimethoate	9 (9.7)	23 (23.9)	13 (18.1)
Fenthion	4 (4.3)	9 (9.4)	10 (13.9)
Others^*a*^	4 (4.3)	8 (8.3)	9 (12.5)

^*a*^Other organophosphoruses insecticides included: phenthoate, diazinon, and profenofos.

**TABLE 2:** Admission Characteristics of Patients Included in the Analysis

		Cohort	RCT Pralidoxime	RCT Placebo
Time since ingestion, h; *n*		3.8 (2.3–5.5); *n* = 91	4.7 (3.3–8); *n* = 86	4.2 (3–6.8); *n* = 71
AChE activity on admission, (mU/µmol Hb); *n*		107 (32–196); *n* = 72	34 (11–68); *n* = 81	33 (7–52); *n* = 64
BuChE activity on admission, (mU/ml); *n*	All patients	121 (31–343); *n* = 92	35 (0–36); *n* = 89	23 (0–166); *n* = 71
	Chlorpyrifos	121 (45–319); *n* = 70	10 (0–74); *n* = 45	20 (0–133); *n* = 34
	Quinalphos	11 (6–82); *n* = 5	10 (0–1149); *n* = 5	10 (5–350); *n* = 5
	Dimethoate	1245 (501–1754); *n* = 9	534 (130–1099); *n* = 23	783 (294–1556); *n* = 13
	Fenthion	10 (4–25); *n* = 4	10 (3–79); *n* = 8	1 (0–24); *n* = 10
	Others^*a*^	124 (35–228); *n* = 4	415 (3–1072); *n* = 8	35 (5-1344); *n* = 9

Data are median (interquartile range). Data were collected on admission to hospital; recruitment occurred soon after. Data were not available for all patients.

Abbreviations: AChE, acetylcholinesterase; BuChE, butyrylcholinesterase; *n*, numbers of cases on which the analysis is based.

^*a*^Other organophosphoruses insecticides included: phenthoate, diazinon, and profenofos.

### 

#### BuChE Activity on Admission

For patients in both cohort and RCT, there were differences in BuChE activity on admission (*p* < .001). Patients with chlorpyrifos poisoning had substantially lower BuChE activity than dimethoate-poisoned patients (Table 2) (*p* < .001). This finding has been previously reported (Eddleston *et al.*, 2008b). Although the number of patients taking fenthion and quinalphos was small, both insecticides also inhibited BuChE to a significantly greater extent than dimethoate (Table 2; *p* = .002 and *p* < .001, respectively).

#### Pralidoxime Regimens and AChE Activity

The pralidoxime regimen used in the RCT (2g loading dose over 20min, followed by a steady infusion of 0.5g/h) produced a measured peak plasma pralidoxime concentration of 250 µmol/l at 1h and a steady state concentration around 100 µmol/l (Eddleston *et al.*, 2009a). Plasma concentrations were not measured in the cohort study due to the intermittent pralidoxime administration. However, the much more rapid bolus administration of 1g in this study (typically over < 1min) would have produced a higher peak pralidoxime concentration, than the 2-g loading dose, that would have rapidly decreased over time (half-life usually < 1h).

**FIG. 2.**
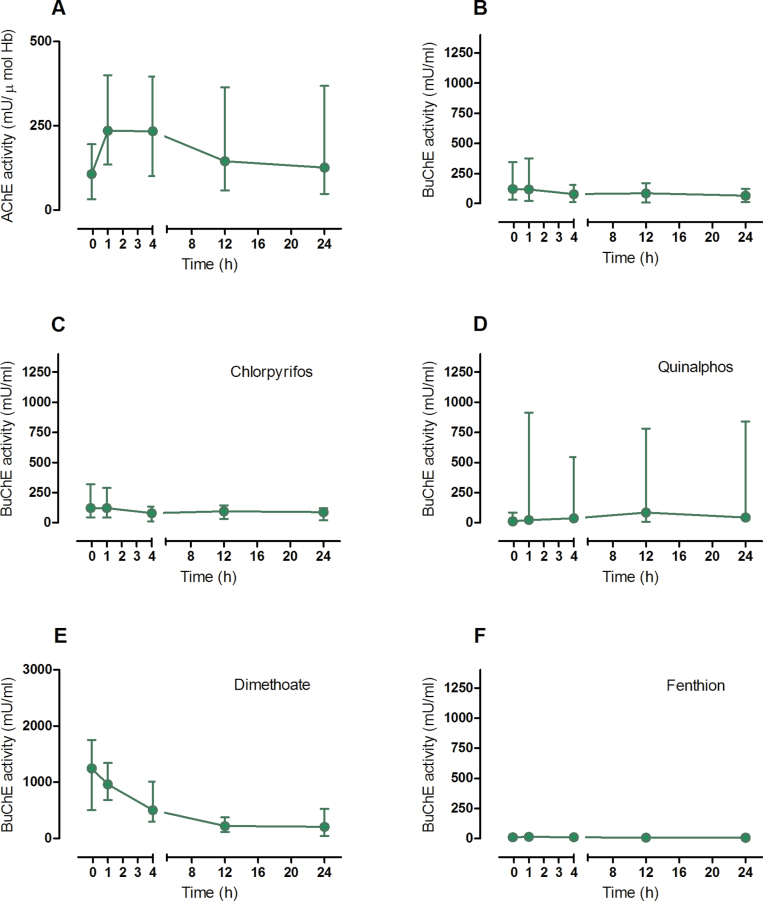
AChE and BuChE activity in poisoned patients receiving bolus pralidoxime (1g bolus every 6 hrs for 24 hrs after admission) (median with interquartile range [IQR]). A, AChE activity for the cohort study patients included in this analysis; B–F: BuChE activities with B, all patients; C, chlorpyrifos; D, quinalphos; E, dimethoate; F, fenthion poisoning. Time = 0 is the time of first pralidoxime administration. Abbreviations: AChE, acetylcholinesterase; BuChE, butyrylcholinesterase.

**FIG. 3.**
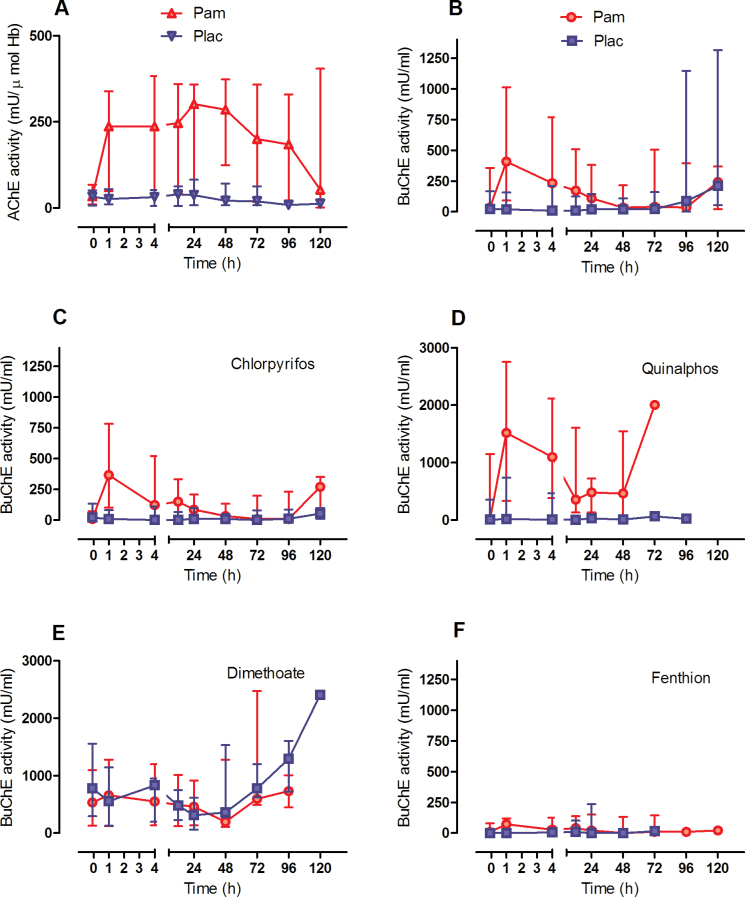
AChE and BuChE activity in poisoned patients receiving pralidoxime by infusion (2g loading dose over 20min followed by 0.5mg/h; circle) or placebo (square) (median with IQR). A, AChE activity for the RCT patients included in this analysis; B–F: BuChE activities with B, all patients; C, chlorpyrifos; D, quinalphos; E, dimethoate; F, fenthion poisoning. Time = 0 is time of first pralidoxime administration. Abbreviations: AChE, acetylcholinesterase; BuChE, butyrylcholinesterase.

Both regimens reactivated AChE (Figs. 2A and 3A) as previously reported (Eddleston *et al.*, 2005, 2009a). Pralidoxime 1 and 2g (followed by 0.5g/h) increased mean AChE activity at 1h by 139 (95% CI 94–184, *p* < .001) and 170 (95% CI 134–205, *p* < .001) mU/µmol Hb, respectively.

#### Effect of Pralidoxime on BuChE Activity

Assessing the total population of cohort patients, treatment with pralidoxime 1g bolus produced no reactivation of BuChE activity at 1h (Fig. 2B; mean difference 55 mU/ml [95% CI −64 to 174, *p* = .36]). Further 6 hourly bolus doses had no apparent effect over the first 24h. Assessing individual OP insecticides, BuChE activity nonsignificantly decreased in dimethoate-poisoned patients in the first hour (Fig. 2E, mean difference −59 mU/ml [95% CI −395 to 278, *p* = .69]). No significant reactivation occurred in chlorpyrifos (Fig. 2C, mean difference 50 mU/ml [95% CI −96 to 195, *p* = .50]), quinalphos (Fig. 2D; mean difference 338 mU/ml [95% CI −567 to 1244, *p* = .36]), or fenthion (Fig. 2F; mean difference 5 mU/ml [95% CI −25 to 34, *p* = .66]) poisoned patients.

Assessing the total population of RCT patients receiving pralidoxime of 2g loading dose over 20min, followed by 0.5mg/h, BuChE over the first hour was significantly reactivated (Fig. 3B, mean difference 416 mU/ml [95% CI 262 to 571, *p* < .001]). The difference was maximal at 1h and decreased up to 48h as BuChE became re-inhibited. This re-inhibition of BuChE—despite the steady infusion of pralidoxime—was quite different from that seen with AChE, which remained activated after initial reactivation with this dose of pralidoxime (Fig. 3A).

BuChE reactivation occurred almost entirely in patients poisoned with diethyl OP insecticides, because reactivation was absent in dimethoate-poisoned (Fig. 3E, mean difference 22 mU/ml [95% CI −142 to 186, *p* = .79]) and fenthion-poisoned (Fig. 3F, mean difference 0 mU/ml [95% CI −59 to 58, *p* = .99]) patients. The level of BuChE reactivation was small in chlorpyrifos-poisoned patients (to 17% of the lower limit of normal, Fig. 3C; mean difference 513 mU/ml [95% CI 310 to 716, *p* < .001]), whereas reactivation in quinalphos-poisoned patients was nonsignificantly greater (to 36% of the lower limit of normal, Fig. 3D; mean difference 1076 mU/ml [95% CI −320 to 2472, *p* = .10]).

#### Variability by Patient

**FIG. 4.**
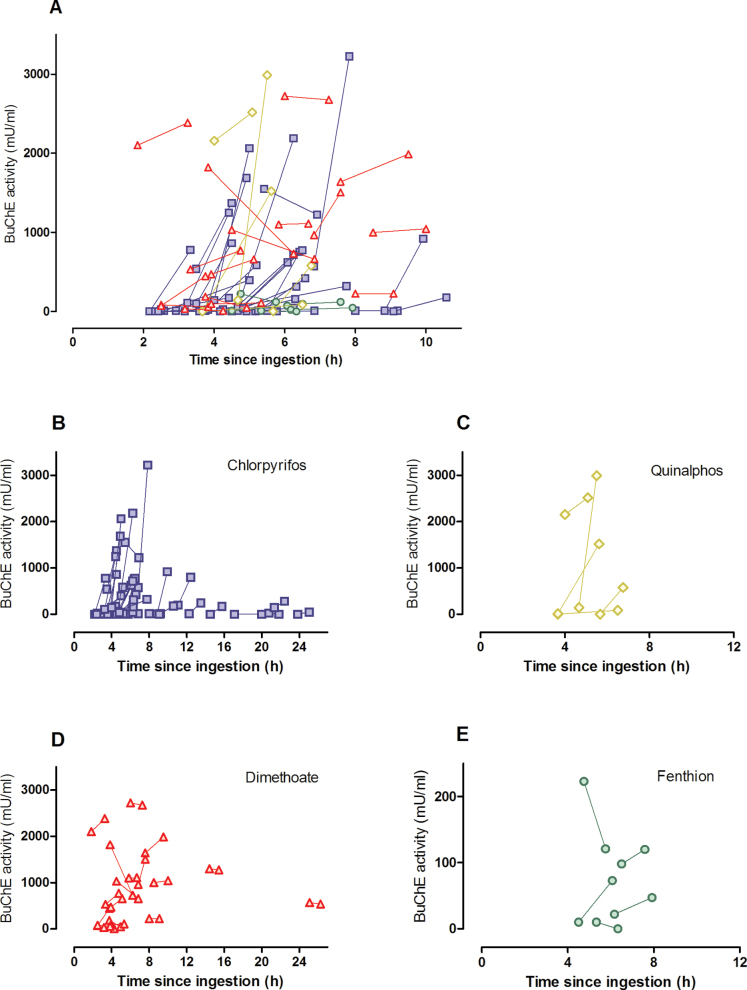
Change in BuChE activity over the first hour from start of pralidoxime treatment in pralidoxime-treated patients in the RCT (2g loading dose over 20min, followed by 0.5g/h) plotted against time since poisoning. The 2 values from each patient are linked with a line. A, all patients; B, chlorpyrifos; C, quinalphos; D, dimethoate; E, fenthion-poisoned patients. Abbreviations: Pam, patients receiving pralidoxime; Plac, patients receiving placebo; BuChE, butyrylcholinesterase; RCT, randomized controlled trial.

**FIG. 5.**
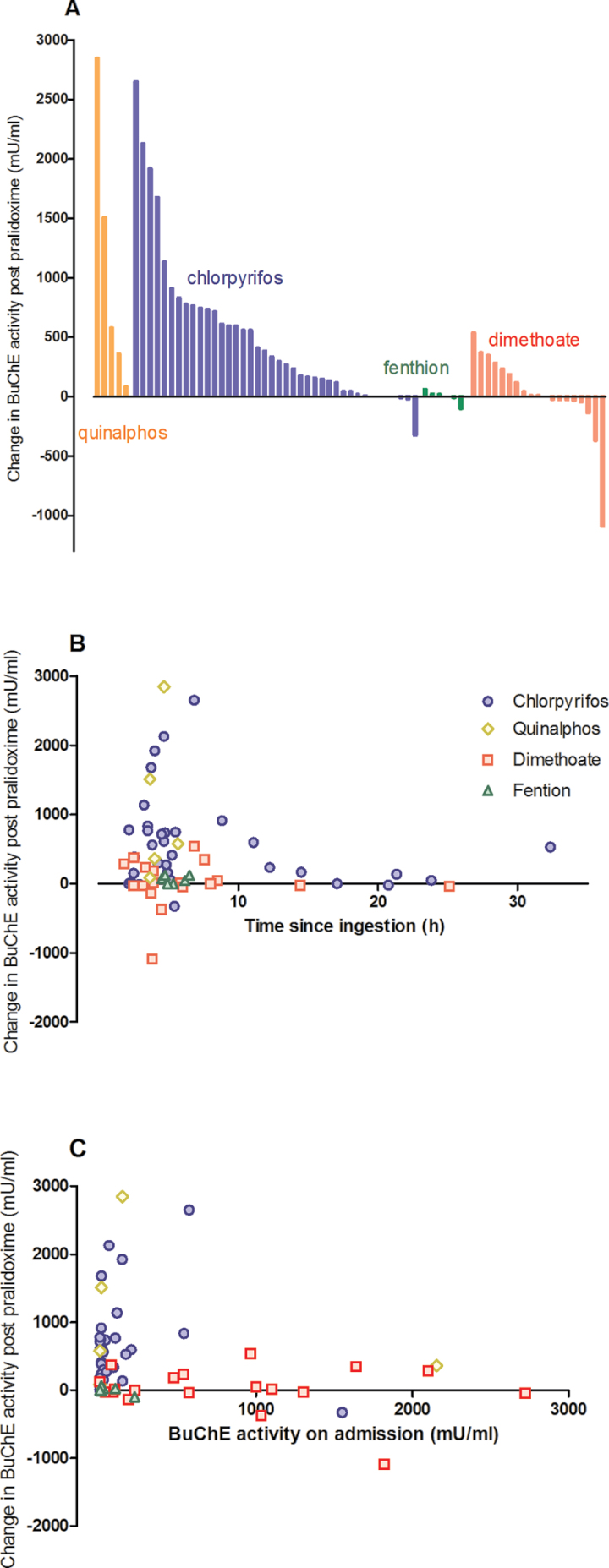
Change in BuChE activity over the first hour from start of pralidoxime treatment in pralidoxime-treated patients in the RCT (2g loading dose over 20min, followed by 0.5g/h). A, change in BuChE for each patient, ordered by size and OP ingested; B, plot of BuChE activity change versus time since ingestion; C, plot of BuChE activity change versus BuChE activity on admission. Chlorpyrifos (circle), quinalphos (diamond), dimethoate (square), and fenthion (triangle). Abbreviations: BuChE, butyrylcholinesterase; OP organophosphorus; RCT, randomized controlled trial.

**FIG. 6.**
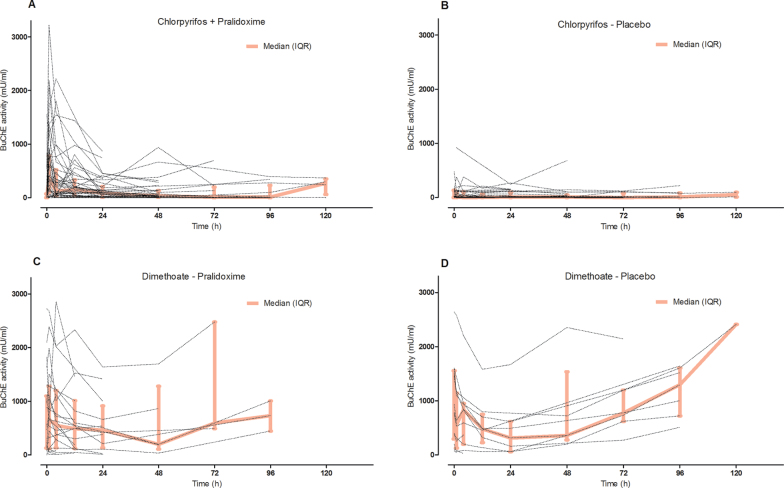
Change in BuChE activity for each RCT patient over time, for the two most common OPs. A, chlorpyrifos with pralidoxime; B, chlorpyrifos with placebo; C, dimethoate with pralidoxime; and D, dimethoate with placebo. Median and IQR are shown by the thick line. Abbreviations: BuChE, butyrylcholinesterase; IQR, interquartile range; RCT, randomized controlled trial.

Looking at individual patients, marked variability occurred within the general pattern of responsiveness to pralidoxime in patients receiving the higher RCT dose (Figs. 4–6). For example, some patients poisoned by chlorpyrifos showed increases of greater than 2500 mU/ml at 1h, whereas others showed further inhibition (Fig 5A). This variability persisted over several days for the 2 most common OP insecticides, chlorpyrifos and dimethoate, with and without pralidoxime (Fig. 6).

The relationship between BuChE reactivation in the first hour and delay to pralidoxime administration postpoisoning or BuChE activity at time of pralidoxime administration was assessed for each OP insecticide. With chlorpyrifos, as expected, reactivation at 1h was inversely related to time since poisoning (Figs. 4B and 5B). For the other OPs, no such relation was apparent although there were few patients with quinalphos or fenthion poisoning. No relationship between BuChE activity at pralidoxime administration and BuChE reactivation at 1h was found (Fig. 5C).

## DISCUSSION

In this study, we have shown that a bolus of pralidoxime chloride 1g does not reactivate BuChE inhibited by WHO class II OPs. In contrast, a 2-g loading dose over 20min, followed by a steady infusion of 0.5mg/h, reactivated diethyl OP-inhibited BuChE although this was not sustained. No reactivation occurred of dimethyl OP-inhibited BuChE. There was marked variation between individuals in how they responded to pralidoxime. These findings extend the results of these previously published cohort and RCT.

BuChE reactivation has been used as a marker of pralidoxime dosing and therefore efficacy in OP-poisoned patients. However, BuChE inhibition is not relevant to the pathophysiology of OP poisoning, and its usefulness would need to be linked to either clinical features or an association with AChE inhibition (if AChE is a good marker, see below). BuChE activity is not closely linked to severity in some forms of OP poisoning, eg, BuChE can be close to zero in patients with few clinical signs following chlorpyrifos poisoning (Eddleston *et al.*, 2008b).

This study shows that BuChE activity after pralidoxime therapy does not closely correlate with AChE activity. BuChE reactivation is much less than AChE reactivation and is not sustained by pralidoxime infusions. AChE activity has been recommended as a useful marker of pralidoxime function (Thiermann *et al.*, 2010). However, recently, several findings have produced doubts about the usefulness of AChE as a marker for WHO class II OP insecticides (Eyer *et al.*, 2010)—a lack of clinical benefit despite clear reactivation of AChE in an RCT (Eddleston *et al.*, 2009a), a lack of correlation with severity in profenofos poisoning (Eddleston *et al.*, 2009b), and a lack of correlation with clinical features in a pig model of dimethoate pesticide poisoning (Eddleston *et al.*, 2012). It is likely to be better to use a clinical marker (such as neurophysiological tests of neuromuscular junction function) than a biochemical marker to follow pralidoxime (or other forms of oxime) efficacy (Thiermann *et al.*, 2010).

We did not measure BuChE aging in these samples. In spite of the decrease in reactivation when the time since ingestion increased, no correlation was found. In addition, it is unlikely that aging was responsible for the very poor reactivation in most patients because the *in vitro* half-life of aging of human BuChE for dimethylated and diethylated enzyme, respectively, is about 3 and 9h (Aurbek *et al.*, 2009) and the median time to presentation of these patients was 3–5h. This would suggest that a median of 50% of BuChE would be available for reactivation with dimethyl OP-poisoned patients and more than this for diethyl OP-poisoned patients.

*In vitro* studies have been done to measure the reactivation of BuChE by pralidoxime for WHO class I and II OP poisonings. Jafari and Pourheidari (2006) showed that pralidoxime 100μM reversed human BuChE inhibition by parathion and by paraoxon by about 50%. Rotenberg *et al.* (1995) showed that obidoxime (175 µg/ml) reactivated BuChE inhibited by chlorpyrifos by 70% and parathion by 90%. In contrast, other *in vitro* studies have shown that neither pralidoxime nor obidoxime can usefully reactivate BuChE inhibited by paraoxon, parathion, or methyl parathion (Aurbek *et al.*, 2009; Musilova *et al.*, 2009). Due to these findings, Aurbek *et al.* (2009) concluded that BuChE activity was inappropriate for monitoring the efficacy of standard doses of pralidoxime after WHO class I OP poisoning.

BuChE assays can be useful for OP poisoning because they may indicate likely exposure and be used to monitor the elimination of the OP (Eddleston *et al.*, 2008a; Kwong, 2002). The liver synthesizes and secretes BuChE continuously; hence, an increase in BuChE activity may indicate the absence of an inhibiting OP in the circulation and the end of a cholinergic crisis (Mason, 2000). This may explain the rise in BuChE activity in patients poisoned by dimethoate (an OP that is rapidly eliminated) after 48h. AChE assays would not be a good marker of OP elimination because reproduction of AChE will occur by erythropoiesis, a slow process with a regeneration of less than 1% AChE of normal each day. Cholinesterase status after OP poisoning is best established by measuring AChE and BuChE activities, re-activatability, and inhibitory activity (a marker of active anticholinesterase in the sample; Eyer, 2003; Eyer *et al.*, 2003).

In summary, we show that pralidoxime-induced reactivation of BuChE is highly variable, according to the dose, OP involved, and the individual poisoned. This indicates that BuChE assays are not useful for monitoring the effect of pralidoxime treatment *in vivo* for poisoning with WHO class II OP insecticides.

## FUNDING

Wellcome Trust (063560); Chief Scientist Office & Scottish Funding Council (Senior Research Fellowship); Wellcome Trust/National Health and Medical Research Council International Collaborative Research Award (071669).
